# Analysis of the Regulatory Science Applied to a Single Portfolio of Eight Biosimilar Product Approvals by Four Key Regulatory Authorities

**DOI:** 10.3390/ph14040306

**Published:** 2021-04-01

**Authors:** Beverly Ingram, Rebecca S. Lumsden, Adriana Radosavljevic, Christine Kobryn

**Affiliations:** 1 Pfizer Inc., Andover, MA 01810, USA; adriana.radosavljevic@gmail.com; 2 Pfizer Inc., Walton Oaks, Surrey KT20 7NS, UK; rebecca.s.lumsden@pfizer.com; 3 Pfizer Inc., Groton, CT 06340, USA; christine.kobryn@pfizer.com

**Keywords:** biosimilars, regulatory, review, approval, clinical, queries, regulatory science

## Abstract

Slow uptake of biosimilars in some regions is often attributed to a lack of knowledge combined with concerns about safety and efficacy. To alleviate physician and patient apprehensions, regulatory reviews from four major regulatory authorities (RAs) (European Medicines Agency, US Food and Drug Administration, Health Canada, and Japan Pharmaceuticals and Medical Devices Authority) across a portfolio of eight biosimilars were analyzed to provide insight into RA review focus and approach. RA queries were evaluated in an unbiased and systematic manner by major classification (Chemistry, Manufacturing and Controls [CMC], nonclinical, clinical or regulatory) and then via detailed sub-classification. There was a consistent, predominant focus on CMC from all RAs. The review focus based on sub-classification of clinical and regulatory queries was influenced by molecular complexity, with significant differences between categories (monoclonal antibody or protein) in the distribution of query topics; specifically, bioanalytical (*p* = 0.023), comparative safety and efficacy (*p* = 0.023), and statutory (including the justification of extrapolation) (*p* = 0.00033). Each biosimilar had a distinct distribution of clinical query topics, tailored to product-specific data. This analysis elucidated areas of heightened RA interest, and validated their application of regulatory science in the evaluation of biosimilar safety and efficacy.

## 1. Introduction

Biosimilars represent an increasingly important option in the delivery of high-quality treatments for patients and offer the potential to address one of the greatest access constraints to biologics globally, namely price [1,2,3]. Since the first biosimilar was approved in 2006 by the European Medicines Agency (EMA) a dedicated regulatory framework for such products has spread rapidly across the world, with biosimilar-specific regulatory paradigms currently established in over 20 countries [4].

The requirement to establish dedicated biosimilar-specific regulatory paradigms by regulatory authorities (RAs) is well documented and is necessary since biosimilars cannot be safely regulated by the pathway used for typical ‘small molecule’ generic drugs [5,6]. The inherent variation of biological systems means that biosimilars cannot be manufactured to be identical to the originator biologic reference product (i.e., reference product) but are instead structurally and functionally “highly similar” [7]. Building and expanding on scientific principles and methodologies established for novel biologics (i.e., concepts outlined in the International Council for Harmonisation Q5E [8]), the EMA issued the first dedicated biosimilar-specific guidance in 2005. This was followed by the World Health Organization (2009), The Japanese Ministry of Health, Labour and Welfare (2009), Health Canada (HC) (2010) and the US Food and Drug Administration (FDA) (2012) (Initial draft overarching guidance was published in 2012; final guidance was published in 2018). Although developed at different times, these guidances share the same fundamental scientific approach to establishing biosimilarity [9,10]. Major regulators such as the FDA, EMA, HC, and the Pharmaceuticals and Medical Devices Authority (PMDA) have leveraged cross-communication, such as health authority cluster meetings, in order to share learning and foster greater consistency, due to the rapid pace at which the regulatory science has evolved [11].

**Figure 1:**
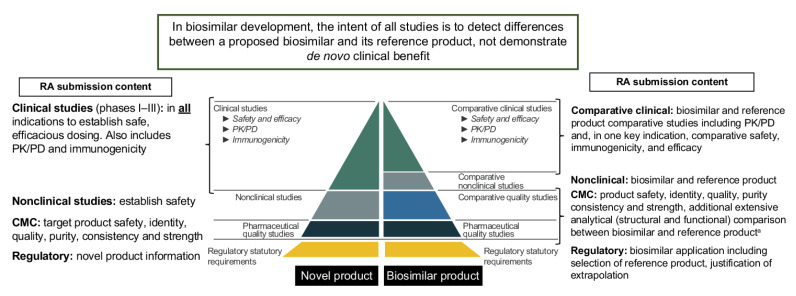
Development Aim and Impact on Regulatory Information for Novel Biologics and Biosimilars (Adapted from Biosimilar Development and Approval in the EU, European Medicines Agency [15]). ^a^ While CMC information for a novel biologic is focused solely on the target product, the corresponding information for a biosimilar is highly comparative, with additional content required focusing on both the biosimilar and the reference product. CMC, Chemistry, Manufacturing and Controls; EU, European Union; PD, pharmacodynamic; PK, pharmacokinetic(s); RA, Regulatory Agency.

The concept of biosimilar development is underpinned by both established scientific knowledge and the application of regulatory science during the assessment by RAs [11,12]. The extent and type of the data required, and the studies conducted during biosimilar development, to meet the regulatory requirements for biosimilarity differ from those required for novel biologics, both in their design and the relative emphasis of contributing parts (Figure 1) [13,14,15]. RAs also have discretion, as per their respective regulatory guidelines, to determine whether some nonclinical and clinical studies are not required; for example, animal studies may be conducted if residual uncertainties remain following the analytical assessment that need to be resolved prior to conducting a comparative clinical trial [16,17,18].

The demonstration of similarity is first and foremost required at a molecular level, by the application of a number of in vitro analytical techniques [19]. These analytical studies are extensive and form the foundation for establishing similarity [13,16,20]. Hence, biosimilar development is focused on the production of a similar molecule to the reference product, analytical (in vitro) assessments that support demonstration of similarity, and manufacturing controls that ensure similarity is maintained [17]. All of these aspects are complemented by a targeted clinical study, sensitive enough with regard to the design, conduct, endpoints and/or population to detect differences should they exist with the reference product [21]. Demonstration of biosimilarity is based on careful consideration of the totality of the information provided [13].

The biosimilar developer can seek approval of their product for other authorized indications of the reference product via extrapolation of similarity. This is a scientific and regulatory principle that is applied without the need to conduct a comparative clinical study in the extrapolated disease indication(s) [22]. Without this facility, biosimilar development would not follow an abbreviated pathway [23]. Extrapolation of efficacy and safety data from one indication to another is not a given; it must be thoroughly scientifically justified, based on data that indicates certain properties of the originator, such as the mechanism of action, PK and immunogenicity, are consistent between the indications [24].

Despite many biosimilars now approved by the EMA [25] and a growing number of biosimilars authorized by the FDA [26], barriers remain to their adoption and use in clinical practice, driven by several issues, including concerns among healthcare providers and patients over their safety and effectiveness [27]. These reservations suggest that gaps may exist between the extent of the evidence required for biosimilars to gain RA approval and the evidence needed to achieve wider acceptance and use by physicians and patients [28]. When the development components and supporting data of a novel biologic and biosimilar are compared (Figure 1), the unique aspects of biosimilar development are revealed as one potential root cause of this gap. The use of an expanded analytical assessment, together with targeted clinical data obtained in a sufficiently sensitive patient population (with justification of extrapolation for additional indications), in place of the more extensive clinical data required for novel biologics, is the foundation of biosimilarity and of the scientific benefit–risk considerations applied by RAs [11].

Pfizer has established a portfolio of biosimilars, which differ in their molecular complexity and span disease indications in inflammation and oncology (including supportive care). Proactive engagement with RAs occurred throughout each product’s development (via advice procedures) to ensure alignment with expectations and requirements. The RA advice from multiple agencies was incorporated into the respective product’s development to inform a global development strategy. This permitted a global dossier preparation and submission approach, whereby the same data for each biosimilar were used to support all submissions (with the inclusion of additional/alternative data to meet a limited number of country-specific requirements). This strategy, and the breadth and extent of regulatory submissions, provides a unique opportunity to analyze the focus of RA review and gain an understanding of the approaches applied by different regulatory bodies in ensuring the requirements for biosimilarity are met. We conducted an analysis of the queries received from multiple RAs in response to license applications for this portfolio of biosimilars. We aimed to bring a greater awareness and appreciation of the RA approach, scientific consistency, and reviewer focus during biosimilar review, to increase confidence in the safety and effectiveness of biosimilars amongst physicians and patients [29].

## 2. Results

A total of 2438 queries were received from the FDA, EMA, PMDA, and HC in relation to 21 applications for the eight biosimilars. Except for two queries relating to legal matters received from the PMDA, all other queries were retained and included in the analysis.

**Figure 2:**
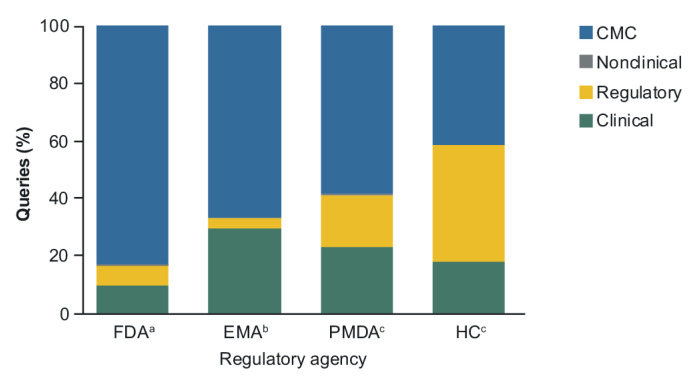
Regulatory Agency Queries Overall (FDA [*n* = 1397]), EMA [*n* = 791], PMDA [*n* = 608], and HC [*n* = 640]) by Major Classification. CMC, Chemistry, Manufacturing and Controls; EMA, European Medicines Agency; FDA, US Food and Drug Administration; HC, Health Canada; PMDA, Pharmaceuticals and Medical Devices Agency. Number of biosimilars: ^a^ *n* = 8, ^b^ *n* = 5, ^c^ *n* = 4.

CMC was the largest category of query assignments received from the FDA (83%), EMA (66%) and PMDA (58%). For HC, 41% of queries were assigned to CMC, which were comparable in number to those assigned to the regulatory category (Figure 2). CMC queries encompassed data supporting the comprehensive in vitro comparative analysis of the biosimilar and its reference product, as well as manufacturing details and quality control aspects, while those assigned to the regulatory category included those focused on the relevance of the reference product, justification of extrapolation of indications, and labeling topics. Nonclinical queries, which related to the limited in vivo studies required, comprised 0.3% or fewer of the overall number of queries received from each RA.

**Figure 3:**
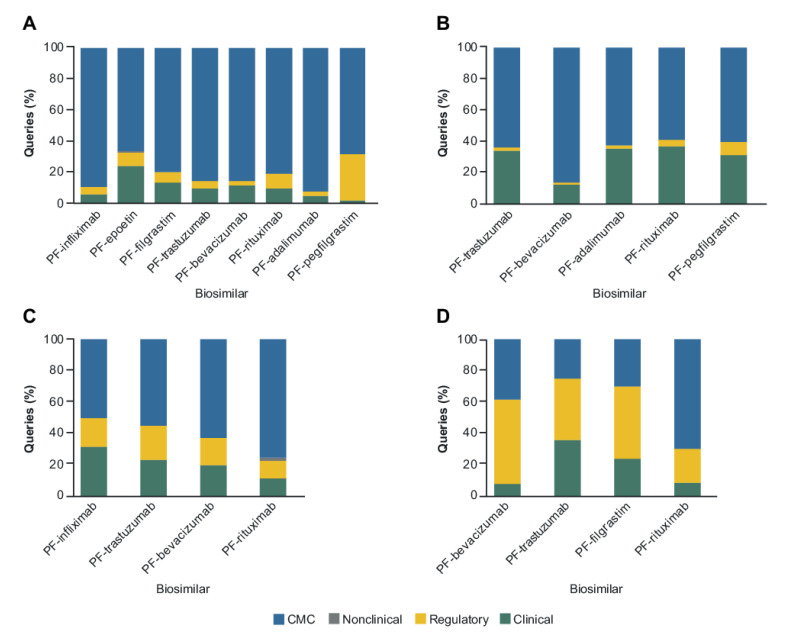
Major Classification Queries by Biosimilar across the (**A**) FDA (*n* = 1397), (**B**) EMA (*n* = 791), (**C**) PMDA (*n* = 608), and (**D**) HC (*n* = 640). CMC, Chemistry, Manufacturing and Controls; EMA, European Medicines Agency; FDA, US Food and Drug Administration; HC, Health Canada; PMDA, Pharmaceuticals and Medical Devices Agency.

A main focus on CMC-related information was also reflected in the queries associated with the individual biosimilars received from the FDA, EMA and PMDA, which was maintained throughout the duration of review period covered by the first and most recent biosimilar to be authorized (Figure 3A–C). Emphasis on the regulatory classification by HC was apparent across all four biosimilars (Figure 3D). Closer evaluation of the queries assigned to this category for HC found that the majority (90%) were related solely to labeling, with CMC queries representing 65% of the overall share when labeling queries were not included. In contrast, both the FDA and EMA directed the lowest proportion of queries towards regulatory topics, comprising 5% and 3%, respectively, of the total queries received from each RA (Figure 2), irrespective of the particular biosimilar (Figure 3A,B).

For the three biosimilars that were assessed by all four RAs (Figure 3A–D), the analysis found a high focus on CMC topics (PF-trastuzumab [Trazimera^™^], 25–86% of queries; PF-bevacizumab [Zirabev^™^], 38–86%; and PF-rituximab, 59–81%). While the FDA raised the highest number of queries overall for each biosimilar, the proportions of queries assigned to the clinical (1–23%) and regulatory (2–30%) categories were relatively low across products with respect to the proportion of CMC queries (67–92%). CMC also represented the most frequent category of queries received from the EMA, with the proportion overall (Figure 2) and by individual product being relatively lower than that seen for the FDA. Amongst the three biosimilars assessed by both RAs, the proportions of CMC queries for PF-bevacizumab were the same for the FDA and the EMA (86%).

### 2.1. CMC Category

**Figure 4:**
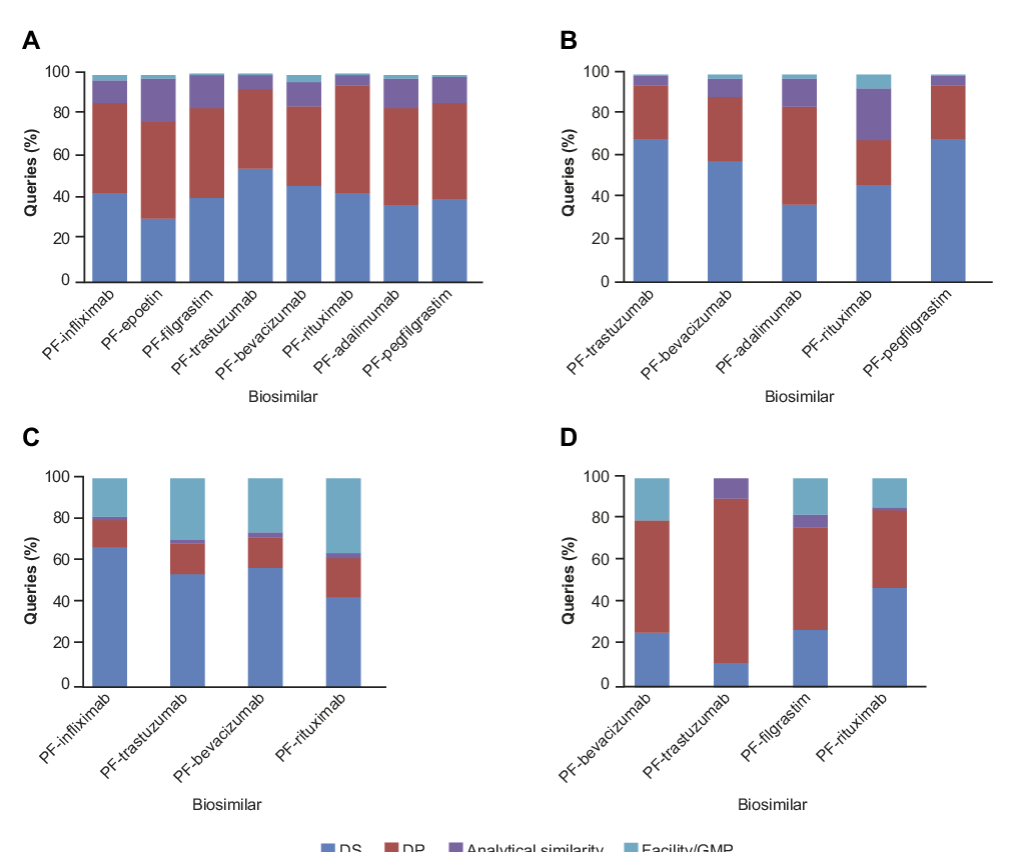
CMC Queries by Biosimilar across the (**A**) FDA (*n* = 2766), (**B**) EMA (*n* = 1134), (**C**) PMDA (*n* = 588), and (**D**) HC (*n* = 318). CMC, Chemistry, Manufacturing and Controls; DP, drug product; DS, drug substance; EMA, European Medicines Agency; FDA, US Food and Drug Administration; GMP, good manufacturing practices; HC, Health Canada; PMDA, Pharmaceuticals and Medical Devices Agency.

Analysis of the CMC queries revealed that the RAs showed a consistent focus on specific aspects, irrespective of molecular complexity or therapy area (Figure 4). The FDA and EMA showed a consistently high focus on both drug substance (DS) (31–54% and 37–69%, respectively) and drug product (DP) content (38–51% and 22–47%, respectively) to a greater extent than analytical similarity (aspects regarding manufacturing and testing control were highly consistently in their inclusion). The FDA were uniquely interested in DP shipping validation information as part of their focus on DP control. Queries related to facilities/good manufacturing practices (GMP) represented <10% (0–3% and 1–7%, respectively) of the overall CMC queries for any individual biosimilar across both the FDA and EMA (Figure 4A,B). In contrast, the queries related to facilities/GMP represented a far higher share of the CMC queries arising from PMDA review of four biosimilars (19–36%) (Figure 4C). Neither the EMA nor the PMDA conducted on-site inspections of manufacturing facilities as part of their review process, in contrast to the approach applied by the FDA and HC. Compared with the other RAs a higher proportion of queries related to DP were received from HC (38–80%), with between 23% and 70% of these being related to sample testing questions (namely detailed queries on how to conduct the analytical methods as well as data interpretation) across the biosimilars assessed. Analytical similarity was generally the least frequent CMC category amongst the HC (0–9%) and PMDA queries (2–3%). Extensive in vitro functional data was submitted and categorized under analytical similarity, which always received close attention by all RAs especially when it related to the product mechanism of action.

### 2.2. Clinical and Regulatory Sub-Classification

**Figure 5:**
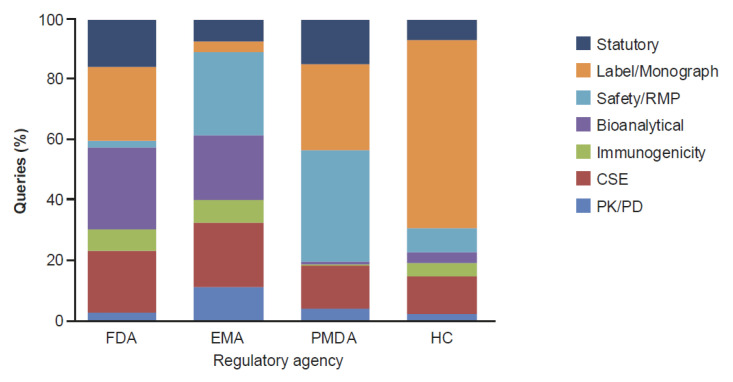
Sub-Classification of Regulatory (Including Labeling) and Clinical Queries by RA (FDA [*n* = 235]), EMA [*n* = 265], PMDA [*n* = 251], and HC [*n* = 377]). CSE, comparative safety and efficacy; EMA, European Medicines Agency; FDA, US Food and Drug Administration; HC, Health Canada; PK, pharmacokinetic; PMDA, Pharmaceuticals and Medical Devices Agency; RMP, risk-management plan.

On sub-classification of the clinical and regulatory queries by RA assigned in the major classification, queries from the FDA and EMA were more focused on bioanalytical aspects than on PK/PD or immunogenicity. A high proportion of those received from HC were assigned to labeling (62%), compared with 29% and 24% on this topic amongst those received from the PMDA and FDA, respectively (Figure 5). Labeling represented <5% of the clinical and regulatory queries received from the EMA. The apparent focus on labeling queries by HC and PMDA was further assessed to identify the share of queries directed towards the presentation of specific biosimilar data in the product label and monograph, or non-data-related queries (including formatting, use of reference product trade name vs. international nonproprietary name vs. biosimilar trade name, etc.). The majority of HC labeling queries were not related to the presentation of biosimilar-specific data in the product label, but on non-data-related queries, with 89% of labeling queries being focused on formatting (Supplementary Figure S1). Likewise, the PMDA reviews overall had only 1 out of 68 (1.5%) labeling queries directed towards biosimilar-specific content, with that query being editorial in nature.

**Figure 6:**
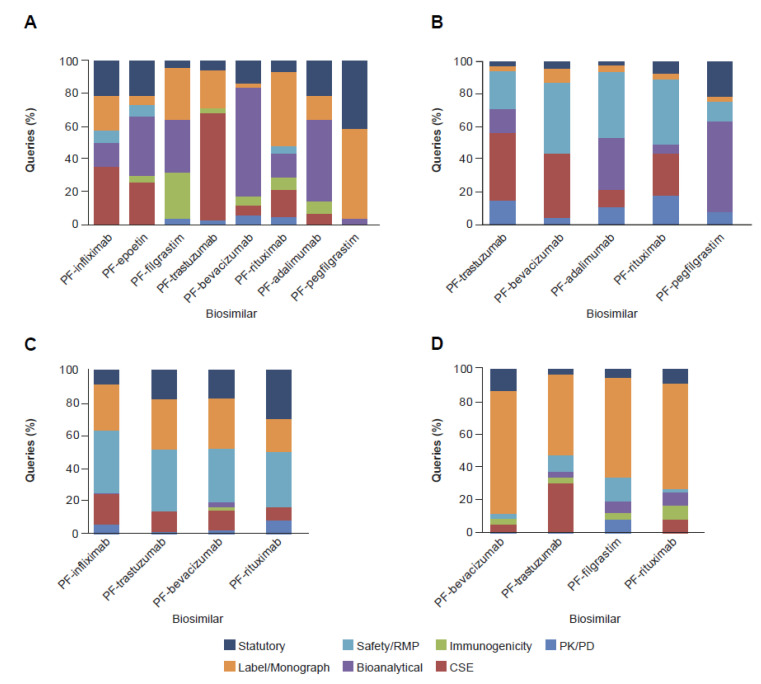
Sub-Classification of Regulatory (Including Labeling) and Clinical Queries by Biosimilar from the (**A**) FDA (*n* = 235), (**B**) EMA (*n* = 265), (**C**) PMDA (*n* = 251), and (**D**) HC (*n* = 377). CSE, comparative safety and efficacy; EMA, European Medicines Agency; FDA, US Food and Drug Administration; HC, Health Canada; PK, pharmacokinetic; PMDA, Pharmaceuticals and Medical Devices Agency; RMP, risk-management plan.

There was no clear focus on any specific clinical and regulatory sub-category when assessing the queries by biosimilar product for the FDA and EMA (Figure 6A,B). Results of the analysis appeared to reflect that safety/risk management plan (RMP) and labeling topics were of consistent interest to PMDA, with the former category comprising 37% of clinical and regulatory queries overall (Figure 6C). The highest frequency of label queries was observed with HC reviews (Figure 6D) comprising 62% of those received across all biosimilars submitted to this RA. Queries directed towards pharmacokinetic (PK)/pharmacodynamic (PD) data were relatively low across all four RAs (Figure 6A–D) and biosimilar products, ranging from 2% to 11%, while bioanalytical assays used to derive the clinical data, comprised 27% and 22% of the clinical and regulatory queries received from the FDA and EMA, respectively (Figure 6A,B). For the three biosimilars (PF-trastuzumab, PF-bevacizumab and PF-rituximab) assessed by all four RAs, each authority raised a different composition of queries on the clinical and regulatory content when presented with essentially the same data (Figure 6A–D).

As indicated in Table 1, the eight biosimilars assessed in this analysis differ in their molecular complexity, which is reflected in their distinct development programs and the data accumulated to support their regulatory approval. They are approved for specific indications within oncology (including supportive care) and inflammatory disease. PF-rituximab is approved for disease indications in both therapy areas.

Across all RAs, neither therapy area [χ^2^ (3, *n* = 1623) = 1.12, *p* = 0.78] nor molecular complexity [χ^2^ (3, *n* = 3436) = 2.14, *p* = 0.54] was found to have a significant relationship with major classification category (Supplementary Figure S2A). The relationship between therapy area and clinical and regulatory sub-classification was also found to be not significant [χ^2^ (6, *n* = 277) = 0.31, *p* = 1.0]. On the other hand, a chi-square test of independence performed to examine the relationship between molecular complexity and clinical and regulatory sub-classification (Supplementary Figure S2B) found the relationship between these variables to be significant [χ^2^ (6, *n* = 1128) = 12.62, *p* = 0.049]. Assessment of the individual clinical and regulatory sub-classifications confirmed that comparative safety and efficacy (CSE) [χ^2^ (1, *n* = 1128) = 5.13, *p* = 0.023], bioanalytical [χ^2^ (1, *n* = 1128) = 5.11, *p* = 0.023], and statutory [χ^2^ (1, *n* = 1128) = 12.88, *p* = 0.00033] query sub-classifications each demonstrated significant relationships with molecular complexity.

## 3. Discussion

In line with the foundational role analytical data plays in the biosimilar development pathway, this analysis established there was a consistently high focus by all RAs on CMC information across all biosimilars, irrespective of their molecular complexity or therapy area (Figure 2).

Analysis of the assignment of queries to CMC categories showed RAs were all highly focused on aspects related to the DS and/or DP including manufacturing/testing controls reflecting the interdependence of this content with analytical similarity. The DS and DP content includes the controls upon which analytical similarity is based; the level of interest by RAs may stem from their aim to establish sufficient rigor will be applied by the manufacturer in maintaining analytical similarity throughout product development. The DS and DP content also includes the control measures to be applied to subsequent commercial manufacturing of the approved biosimilar, and again, review focus on this aspect would ensure similarity should be maintained in the future. The content of the DS and DP release specifications was an area of focus for all RAs, although the attributes and expectations were not identical. Despite the different queries received, the RA intent was clearly to ensure analytical similarity was measured and controlled to meet high expectations.

From the sub-classification of the clinical and regulatory queries there was a relatively greater focus on bioanalytical than on PK/PD or immunogenicity aspects by the FDA and EMA compared with the other RAs. This review approach was applied across all submitted products but not at consistent levels, suggesting it may have been influenced by individual reviewer preference. However, it should be noted that interrogation of the bioanalytical methods by reviewers can be considered an indirect assessment of the validity of the clinical (i.e., PK/PD or immunogenicity) data and this approach may be reflected in the findings for the FDA and EMA. During review, HC typically request visibility of FDA and EMA queries, if available, which may allow them to focus their review elsewhere on elements related to national regulatory (labeling) requirements. In the present analysis, based on the CMC categorization, the PMDA and HC both issued a higher proportion of queries related to facilities/GMP, compared with FDA and EMA, which is most likely due to differences in approval procedures rather than fundamental differences in GMP expectations. Both the FDA and HC review procedures can include site inspections; this only occurs during EMA and PMDA application assessments if a site has not previously been inspected within an acceptable time frame. HC was the only RA of the four covered in this analysis that routinely conducted sample testing as part of their review, which required DP samples and information on the analytical testing method to be provided. The high proportion of DP-related queries issued by HC amongst the CMC categorization across biosimilars could be attributed to sample testing activities, including those related to the transfer of analytical testing methods.

One area that received little attention from the RAs was nonclinical information, comprising <0.3% of queries in our analysis, which is consistent with the less significant role such studies play in biosimilar development compared with a novel biologic. It also supports the ongoing regulatory focus on the principle of the 3Rs (Reduce, Replace, Refine) that has been applied in updates to the earlier biosimilar guidance in some regions [16]. The low number of nonclinical queries received from the RAs that require animal studies (i.e., FDA, PMDA) supports the view that little or no nonclinical information should be necessary for demonstrating biosimilarity [30].

The proportion of clinical queries overall was low compared with requests for CMC information. Differences in the distribution of CMC and clinical queries between RAs is not unexpected and may reflect the experience of the RA authority or reviewer with biosimilars, their expectations, and/or discrete approach to reviewing data. Analysis of the query topics within the clinical sub-classification demonstrated alignment in the focus of the reviews and the principles of the scientific guidance for biosimilars across all four RAs (Figure 1). Analysis of the distribution of clinical queries following sub-classification revealed that the RA questions were tailored to individual biosimilars. Moreover, no prescribed review approach was evident from any of the RAs, with a different composition and distribution of queries being received on clinical topics for each biosimilar. Based on sub-classification of the clinical queries, there was no evidence that the tailored RA review approach was influenced by the therapy area (oncology or inflammation) of the biosimilar. However, the molecular complexity of the biosimilar showed significant association with queries sub-classified to bioanalytical, CSE, and statutory topics (which encompassed biosimilar-specific requirements, including the justification of reference product selection and extrapolation of indications).

Amongst the clinical and regulatory queries following sub-classification, a relatively low proportion were directed towards PK/PD data for all four RAs (Figure 6A–D). This finding was somewhat surprising since comparative PK studies are considered a key component of biosimilar development, due to their role in addressing residual uncertainties arising from the analytical assessment and in establishing there will be no clinically meaningful differences between the proposed biosimilar and reference product. They are also key to guiding the requirement for, and nature of, subsequent comparative clinical studies [31]. The proportion of PK/PD queries was unaffected whether the study was conducted in patients (such as for PF-rituximab [32]) or in healthy subjects (PF-infliximab [Ixifi^™^] [33], PF-epoetin [34,35], PF-filgrastim [36], PF-trastuzumab [37], PF-bevacizumab [38], PF-adalimumab [Abrilada^™^/Amsparity] [39], PF-pegfilgrastim [Nyvepria^™^] [40]). The generally low focus on PK information across RAs may reflect the proactive engagement of the applicant with the relevant RA during the study design process, the transparency of the data disclosure and interpretation. The approach to review of the clinical information was often two-pronged; indirect, via queries related to bioanalytical assays that supported the clinical study conclusions (PK/PD, CSE, and immunogenicity), and direct, with questions focused on the clinical data. In particular, the FDA and EMA appeared to favor focusing on bioanalytical assays as an indirect assessment tool to complement assessment of the data generated in clinical studies (PK/PD, CSE, and immunogenicity) (Figure 5). In the case of the FDA this included routinely requesting internal method validation reports and sample management records.

In general, neither the EMA nor the FDA allow inclusion of clinical data for the biosimilar on the product label, but instead use the data already provided in the reference product label [41,42]. In contrast, the PMDA and HC permit certain data for the biosimilar generated in comparative clinical trials to be included in the label [43,44]. It might be expected that these differences between RAs in relation to inclusion of data in the product label would be reflected in the distribution of the labeling and statutory topics following sub-classification of the regulatory and clinical queries. While our analysis found that both HC and the PMDA placed a greater focus on labeling queries compared with the FDA and EMA, in-depth analysis of their labeling questions determined that biosimilar data-related queries comprised only 11% and 2% of the overall labeling queries received from HC and the PMDA, respectively. This suggests that there may be opportunity for RAs to provide more operationally focused labeling guidance for biosimilars to reduce their effort and resources on raising queries on this topic.

The biosimilar guidance and regulatory requirements for the four RAs covered by this analysis were largely aligned, with only minimal divergence to meet country-specific content requirements. This allowed for submission of consistent content across the different RAs. Each RA showed a broadly similar approach in implementing their guidance, which was evident in their high focus on CMC content (ranging, on average from 41–83%), minimal nonclinical queries (<0.3%), and between 17% and 59% of queries directed to clinical and regulatory topics combined. Comparison of the clinical and regulatory sub-classification queries of the three biosimilars assessed by all four RAs suggested a tailored approach to their review, focused on different topics and with varying frequency across RAs for each biosimilar. Despite aligned guidance and shared high-level expectations across RAs on biosimilar development, the differences in the review focus highlighted here may reflect RA-specific guidance implementation approaches with regard to sub-classification content. For the biosimilars assessed by more than one RA, while their review decision was the same, with approval granted for all requested indications via the justification of extrapolation, their approach to data review and assessment of benefit–risk differed. No common review strategy was observed across the four RAs; however, the approach for each RA reflected a robust assessment of data, and the justification of that data to support biosimilarity and extrapolation of indications. Interestingly, while the RAs were equally effective, the level of review of the sub-classification topics may reflect their differing implementation of the biosimilarity concept.

The biosimilars assessed in this analysis formed a single portfolio of products developed following a global strategy, guided by proactive RA engagement, whereby the same data for each biosimilar were used to support all submissions. Our analysis revealed that the RAs do not have a pre-set approach to data review and their focus is influenced by the individual submission data per product. Although we applied the same global development strategies, the data provided was unique to each product. Therefore, it is anticipated that when assessing other biosimilars of the same reference products covered in our analysis, RAs will focus on the same topics (according to the classification and categorization system used here).

Not all biosimilars were submitted to all RAs. The data submitted to the RAs reflected product-specific information and the particular studies conducted during the biosimilar development program (e.g., comparative PK/PD and immunogenicity for PF-filgrastim and PF-pegfilgrastim were derived from studies conducted in healthy volunteers, in lieu of CSE; data for delivery by autoinjector formed part of the submission package for PF-adalimumab).

The volume of queries served solely as a proxy measure of areas of the biosimilar product dossier that received attention by the RAs. It was not possible to weight the queries to reflect the importance of the questions to the regulators or the complexity of the response required.

## 4. Materials and Methods

From 2017 to November 2020, 21 regulatory submissions for market authorization were undertaken for eight biosimilars in one or more of the major domains comprising the USA, EU, Japan, or Canada, in advance of cascading to further submissions globally. Contemporaneous submissions of the same data content for each biosimilar allows comparison of the review approaches between four RAs: US FDA, EMA, PMDA, and HC. These RAs were selected due to the consistency of their biosimilar guidance and their regulatory requirements, as well as the leading role their guidance has played in shaping regulatory expectations in other countries.

Details of the biosimilars included are provided in Table 1, together with the approval dates in these four RA domains. To ensure comparisons were based on consistent submission content and time frame, EMA approvals of the filgrastim (PF-filgrastim; Nivestim) and epoetin (PF-epoetin: Retacrit^®^) biosimilars in 2010 and 2007, respectively, were excluded from this analysis. The reference products, Neupogen^®^ and Epogen^®^/Procrit^®^, respectively, were developed to include several different aspects in the US and EU (e.g., strength and presentation), and the biosimilars were developed to align with the EMA-approved reference products. As a result, the differences in submission information between EU and US meant that direct comparison could not be made.

Queries received during the course of review of all eight biosimilars by the four RAs were collated and each was categorized retrospectively by the authors using the methods of classification outlined below. Any uncertainties in category assignments were resolved by author calibration meetings. In instances where a query included subsidiary questions (e.g., query X, parts i-v) the main query and subsidiary components were counted separately. In some cases, a query related to a single topic, while others covered multiple topics (e.g., one CMC query could have included aspects that related to both DS and DP). In such instances, a single question could be assigned to more than one sub-classification category.

### 4.1. Major Classification

Initially, queries were assigned to one of four major categories: CMC, nonclinical, clinical, and regulatory (encompassing all statutory requirements such as labeling and justification of extrapolation of indications) (Table 2). The relative frequencies of each category were determined as a percentage of the overall number of queries received from each RA and for each biosimilar. There were relatively few queries assigned to the non-clinical category. Therefore, queries assigned to this category were not sub-classified further.

### 4.2. CMC Sub-Classification

All queries assigned to the CMC category in the major classification were further assigned to four CMC categories according to the criteria in Table 2. Since individual CMC queries may have not have been related to a single topic they could be assigned to more than one CMC category. Relative frequencies of each topic were determined as a percentage of the total volume of CMC queries by RA and by biosimilar.

### 4.3. Clinical and Regulatory Sub-Classification

All queries that were assigned to the clinical and regulatory categories in the major classification were further sub-classified to one of seven categories according to the assignment criteria in Table 2. Relative frequencies of each category were determined as a percentage of the total number of queries received from each RA.

Labeling queries received from HC and PMDA were further subdivided by assignment to either ‘data’ (queries related to the presentation of biosimilar data from comparative clinical studies) or ‘format’ (queries related to label text and unrelated to biosimilar clinical data).

### 4.4. Statistical Analysis

Chi-square tests of independence were performed between molecular complexity (monoclonal antibody [MAb] or protein) and therapy area (oncology or inflammation) versus major query classification for each RA. The rituximab biosimilar (PF-rituximab; Ruxience^™^) was included under inflammation and oncology therapy areas in this analysis since it is approved for disease indications in both.

Chi-square tests of independence were also performed on the basis of molecular complexity and query sub-classification, as well as on the basis of therapy area and query sub-classification.

## 5. Conclusions

Analysis of the focus of the FDA, EMA, HC, and PMDA review of the biosimilars described here gives an indication of the practical application of the regulatory science underpinning the robust regulatory standards that exist in the countries and region served by these RAs. The distinct distribution of queries received for three biosimilars assessed by all four RAs may reflect a different approach in assessing benefit–risk, while still ultimately reaching the same regulatory decision.

Analysis of the focus of RAs on specific query topics identified areas of heightened interest and gave some insight as to their significance. When provided with essentially the same data, aside from country-specific content, all four RAs focused primarily on CMC-related topics, irrespective of the molecular complexity or therapy area of the biosimilar. The level of focus on CMC information was consistent with the fundamental importance of data in this domain to the demonstration of similarity, as the basis for extrapolation of indications, and to the controls applied to biosimilar manufacturing and testing.

The clinical and regulatory data review was tailored and product-specific, irrespective of therapy area, but the focus of the queries based on their sub-classification was significantly associated with the category of molecular complexity. Nevertheless, the proportion of queries on clinical topics overall was relatively low, confirming that the information from clinical studies is deemed by RAs to be largely supportive in demonstrating biosimilarity. The greatest area of RA focus was consistently placed on the assessment of data that represented the most sensitive information in the demonstration of biosimilarity, namely CMC, and the justification for extrapolation of indications.

## Figures and Tables

**Table 1:** Biosimilars and Approval Dates.

Biosimilar		Regulatory Agency, Approval Date				Reference Product, Trade Name (INN)	Molecule Complexity	Therapy Area
Product	INN (Trade Name), US/RoW	FDA	EMA	PMDA	HC			
PF-filgrastim	filgrastim-aafi (Nivestym^®^)/ filgrastim (Nivestim) ^a^	20 July 2018	8 June 2010 ^e^	− ^d^	20 April 2020	Neupogen^®^ (filgrastim)	Protein	Oncology
PF-epoetin	epoetin alfa-epbx (Retacrit^®^) ^b^	15 May 2018	− ^d^	− ^d^	− ^d^	Epogen^®^/Procrit^® g^ (epoetin alfa)	Protein	Oncology
PF-epoetin	epoetin zeta (Retacrit^®^) ^b^	− ^d^	18 December 2007 ^e^	− ^d^	− ^d^	Epogen^®^/Procrit^® g^ (epoetin alfa)	Protein	Oncology
PF-rituximab	rituximab-pvvr/ rituximab (Ruxience™)	23 July 2019	1 April 2020	20 September 2019	4 May 2020	Rituxan^®^ (rituximab)	MAb	Oncology, inflammation ^h^
PF-trastuzumab	trastuzumab-qyyp/trastuzumab (Trazimera™)	11 March 2019	26 July 2018	21 September 2018	15 August 2019	Herceptin^®^ (trastuzumab)	MAb	Oncology
PF-bevacizumab	bevacizumab-bvzr/bevacizumab (Zirabev™)	28 June 2019	14 February 2019	18 June 2019	14 June 2019	Avastin^®^ (bevacizumab)	MAb	Oncology
PF-pegfilgrastim	pegfilgrastim-apgf/pegfilgrastim (Nyvepria™)	11 June 2020	20 November 2020	− ^d^	− ^d^	Neulasta^®^ (pegfilgrastim)	Protein	Oncology
PF-adalimumab	adalimumab-afzb (Abrilada™)/ adalimumab (Amsparity) ^c^	18 November 2019	13 February 2020	− ^d^	− ^d^	Humira^®^ (adalimumab)	MAb	Inflammation
PF-infliximab	infliximab-qbtx/infliximab (Ixifi™)	13 December 2017	Divested ^f^	2 July 2018	− ^d^	Remicade^®^ (infliximab)	MAb	Inflammation

^a^ Known the US as Nivestym^®^ and in the RoW as Nivestim. ^b^ Known in the US and in the RoW as Retacrit^®^, with the products designated by the INNs epoetin alfa-epbx and epoetin zeta, respectively. ^c^ Known as Abrilada™ in the US and as Amsparity in the EU. ^d^ Not all biosimilars have been submitted in all domains. Some applications are ongoing. ^e^ Excluded from the analysis as the reference product was developed to include several different aspects in the US and EU (e.g., strength and presentation). ^f^ Licensing by the EMA was divested by Pfizer in February 2016. ^g^ Market authorization for reference epoetin alfa is held by two companies (Epogen^®^; Amgen Inc., Thousand Oaks, CA, USA and Procrit^®^; Janssen Products, LP, Horsham, PA, USA). ^h^ Approved for the inflammatory disease indications of granulomatosis with polyangiitis and microscopic polyangiitis in the US; not approved for rheumatoid arthritis in the US. EMA, European Medicines Agency; FDA, US Food and Drug Administration; HC, Health Canada; INN, international nonproprietary name; MAb, monoclonal antibody; PMDA, Pharmaceuticals and Medical Devices Authority; RoW, Rest of the World.

**Table 2:** Query Classification and Category Assignment Criteria.

Classification Level	Category (Assignment Criteria)			
*Major*	*CMC* (all aspects of drug substance [DS] and drug product [DP] manufacturing, testing, control and analytical similarity [including in vitro functional analysis])	*Nonclinical* (in vivo animal studies and comparative toxicokinetics between biosimilar and reference product)	*Clinical* (all clinical studies including PK/PD studies in healthy volunteers or patients, comparative safety and efficacy, where appropriate, and immunogenicity)	*Regulatory* (statutory requirements of the submission including prescribing information, selection of reference product, justification of extrapolation, and regulatory procedural topics)
*Sub-classification*	*DS* (DS development, manufacture, control, storage, transportation and stability) *DP* (DP development, manufacture, control, storage, transportation and stability, including queries related to product testing activities [e.g., product sample testing for HC]) *Analytical similarity* (analytical similarity studies, data, and interpretation [including in vitro functional analysis]) *GMP/Facility* (GMP status of facilities involved in the development, manufacturing, and testing of the DS and DP, including queries issued following facility inspections conducted as part of an application (e.g., HC On-Site Evaluation and FDA pre-approval inspection)		*Bioanalytical* (assays used to generate and assess clinical data [all clinical studies]; this included the assay development, validation/qualification, and sample preparation across all clinical studies) *PK/PD* (data derived from the comparative PK/PD modeling) *Immunogenicity* (immunogenicity information derived from the clinical studies) *Comparative Safety and Efficacy (CSE)* (comparative study, generally conducted in only one clinical indication) *Safety/RMP* (safety, including the RMP where applicable [e.g., EMA, PMDA, HC] and pharmacovigilance) *Label/Monograph* (product information [e.g., US PI, EU SmPC, including the Canadian-specific product monograph]) ^a^ *Statutory* (justification of extrapolation, reference product selection, and general regulatory procedural topics [e.g., trade name approval])	

^a^ Queries received from HC and PMDA assigned to labeling/monograph sub-class were further subdivided by assignment to either data (queries related to the presentation of biosimilar data from comparative clinical studies) or to format (queries related to label text and unrelated to biosimilar clinical data). CMC, Chemistry, Manufacturing and Controls; EMA, European Medicines Agency; EU, European Union; FDA, US Food and Drug Administration; GMP, good manufacturing practice(s); HC, Health Canada; PD, pharmacodynamic(s); PK, pharmacokinetic(s); PMDA, Pharmaceuticals and Medical Devices Authority; RMP, risk management plan; SmPC, Summary of Product Characteristics; US PI, United States Prescribing Information.

## Data Availability

The data are contained within the article or supplementary material.

## Supplementary Materials

The following are available online at https://www.mdpi.com/article/10.3390/ph14040306/s1, Figure S1: Sub-classification of Labeling and Monograph Queries for HC, Figure S2: Major Classification Queries and Sub-classification of Regulatory (Including Labeling) and Clinical Queries by Molecular Complexity for all RAs, Table S1: Relationship of therapy area and molecular complexity with major classification, and clinical and regulatory sub-classification.
