# Supplementary material for: Analysis of the Regulatory Science Applied to a Single Portfolio of Eight Biosimilar Product Approvals by Four Key Regulatory Authorities
Source: Pharmaceuticals (Basel). 2021 Apr 1;14(4):306. doi: 10.3390/ph14040306 (PMC8067310; doi:10.3390/ph14040306)
Supplement: Supplementary file 1 [file pharmaceuticals-14-00306-s001.pdf]

Supplementary Materials

Analysis of the Regulatory Science Applied to a Single Portfolio of Eight Biosimilar Product Approvals by Four Key Regulatory Authorities

Beverly Ingram [1,*], Rebecca S. Lumsden [2], Adriana Radosavljevic [1] and Christine Kobryn [3]

[1] Pfizer Inc, Andover, MA 01810, USA; adriana.radosavljevic@gmail.com
[2] Pfizer Inc, Walton Oaks, Surrey KT20 7NS, UK; rebecca.s.lumsden@pfizer.com
[3] Pfizer Inc, Groton, CT 06340, USA; christine.kobryn@pfizer.com
* Correspondence: bev.ingram@pfizer.com; Tel.: +1-978-247-4558

Supplementary Figure S1. Sub-classification of Labeling and Monograph Queries for HC (n = 235).

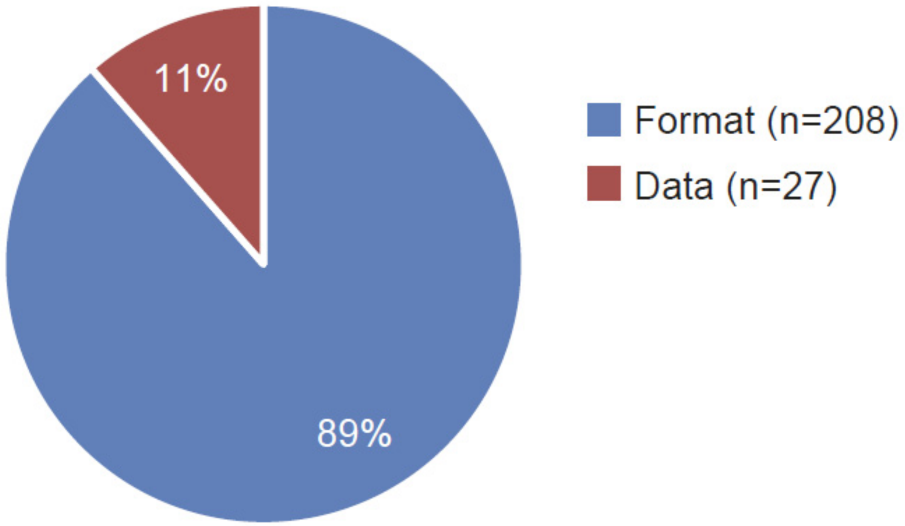

Abbreviations: HC, Health Canada.

Supplementary Figure S2. Major Classification Queries (**A**) and Sub-classification of Regulatory (Including Labeling) and Clinical Queries (**B**) by Molecular Complexity (MAb [n = 2811];[a] protein [n = 625][b]) for all RAs

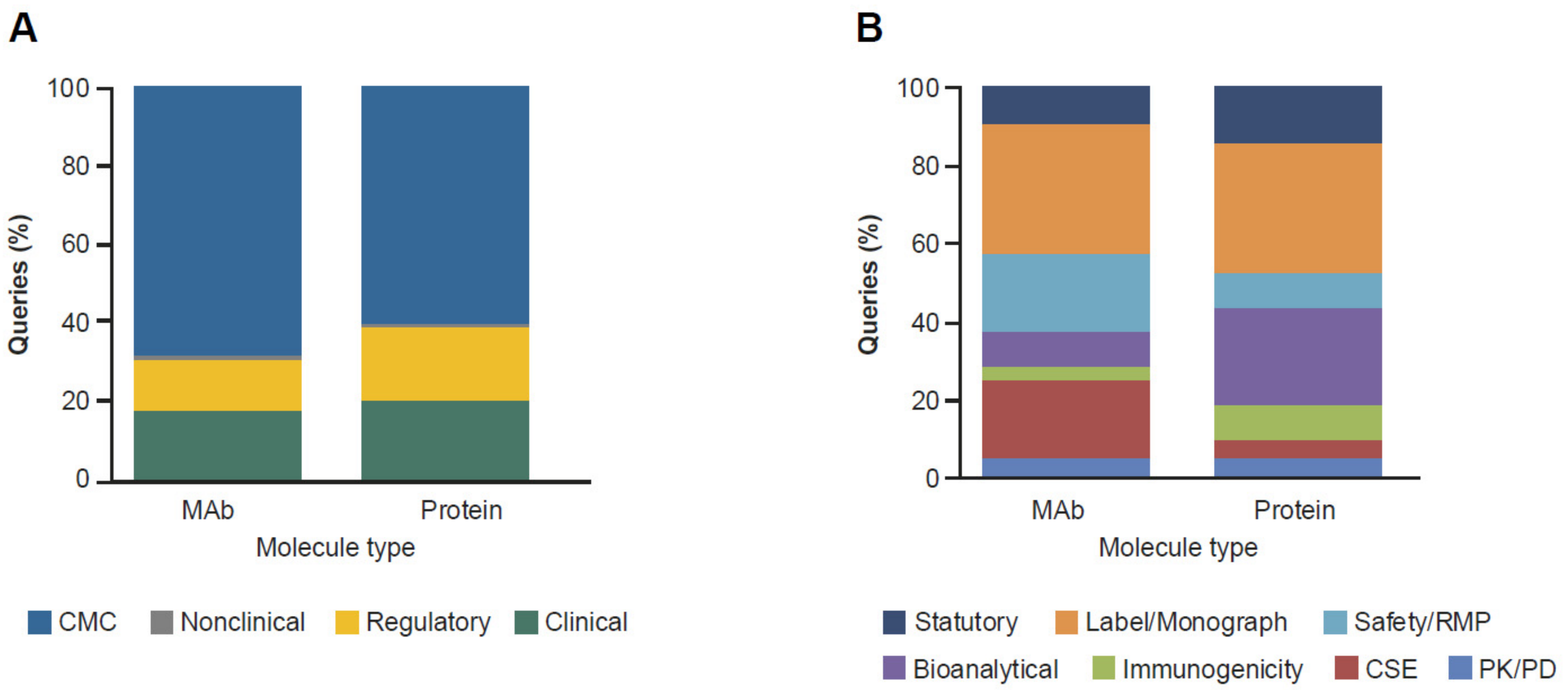

Abbreviations: CMC, Chemistry, Manufacturing and Controls; CSE, comparative safety and efficacy; MAb, monoclonal antibody; PK, pharmacokinetic; RA, regulatory authority; RMP, risk-management plan.

[a] PF-adalimumab, PF-bevacizumab, PF-infliximab, PF-rituximab, PF-trastuzumab; [b] PF-epoetin, PF-filgrastim, PF-pegfilgrastim.

Supplementary Table S1. Relationship of therapy area and molecular complexity with major classification, and clinical and regulatory sub-classification**.**

Variable		Clinical	Regulatory	Nonclinical	CMC	n	χ^2 (df)	*p*-value
Therapy area	Inflammation (n=8)	37	33	1	475	1623	1.12 (3)	.78
	Oncology (n=17)	124	83	2	868			
Molecular type	MAb (n=16)	507	377	3	1924	3436	2.14 (3)	.54
	Protein (n=5)	128	116	1	380			

Variable		PK/PD	CSE	Immunogenicity	Bioanalytical	Safety/ RMP	Label/ Monograph	Statutory	n	χ^2 (df)	*p*-value
Therapy area	Inflammation (n=8)	2	13	4	15	3	24	9	277	0.31 (6)	1.0
	Oncology (n=17)	6	43	15	55	5	52	31			
Molecular type	MAb (n=16)	43	175	33	76	180	294	83	1128	12.62 (6)	.049
	Protein (n=5)	12	12	21	61	22	80	36			

Abbreviations: CMC, Chemistry, Manufacturing and Controls; CSE, comparative safety and efficacy; df, degrees of freedom; MAb, monoclonal antibody; PD, pharmacodynamics; PK, pharmacokinetic; RA, regulatory authority; RMP, risk-management plan.
